# Surface interaction between phyllosilicate particles and sustainable polymers in flotation and flocculation

**DOI:** 10.1039/d1ra07928j

**Published:** 2022-01-28

**Authors:** Nahid Molaei, Mohammad Shoaib, John Forster, Shaihroz Khan, Omar Bashir Wani, Erin R. Bobicki

**Affiliations:** Department of Materials Science and Engineering, University of Toronto Toronto ON Canada erin.bobicki@utoronto.ca; Department of Chemical Engineering and Applied Chemistry, University of Toronto Toronto ON Canada

## Abstract

Non-renewable chemical reagents are commonly used as dispersants or flocculants of phyllosilicate clay particles in several industrial fields such as water/wastewater treatment, food production, papermaking, and mineral processing. However, environmentally benign reagents are highly desired due to the non-biodegradability and negative impacts of synthetic reagents on aquatic life. In this work, the dispersion and flocculation behavior of sustainable polymers (anionic and cationic biopolymers) sourced from proteins and polysaccharides were studied in serpentine phyllosilicate suspensions using the following bench-scale tests: zeta potential, microflotation, settling and turbidity, and isotherm adsorption using total organic carbon. The anionic polysaccharide-based biopolymer pectin acted as a switchable biopolymer for serpentine. That is, it could switch from being an efficient flocculant at pH 7 to an effective dispersant at pH 10.

## Abstract 2

Biopolymers with different backbones have the potential to disperse the phyllosilicate particles in flotation or release the water trapped within tailing particles in flocculation and could decrease environmental problems of conventional reagents.

## Graphical abstract

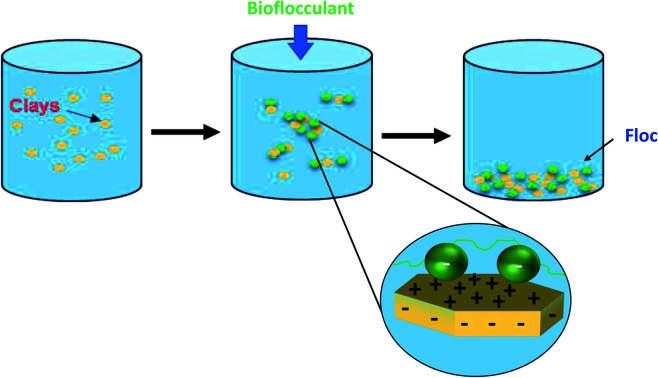

## 1. Introduction

In the face of grade depletion of ores containing valuable minerals, mineral processing operations must perform energy-intensive grinding, which generates significant amounts of fine and ultrafine gangue particles containing phyllosilicate clay minerals.^1,2^ Phyllosilicates are comprised of layered silica tetrahedral sheets and alumina or magnesia octahedral sheets in varying proportions.^3–6^ They can adhere to the surfaces of target minerals during flotation, changing froth stability and rheology and thereby reducing the grade and recovery of valuable minerals.^7,8^ Physical and chemical strategies to counteract the negative effects of phyllosilicate clays have led to overconsumption of some dispersants and depressants, lower pulp solid concentrations, high water consumption, and the need for intense agitation prior to flotation to lower viscosities. Fine phyllosilicate particles are also problematic in mine tailings, thickening operations. Fine and ultrafine charged particles remain suspended in tailings due to repulsive forces between similarly charged particles. Dewatering techniques are required to separate solids and liquids in tailings for water recovery and land reclamation.^9,10^ Coagulation using inorganic salts followed by flocculation with synthetic polymers efficiently removes charged fine particles from water by reducing the repulsive interparticle forces and neutralizing the particle charge, thus allowing particles to associate and form flocs.^11^

Conventional phyllosilicates mitigation methods in flotation and flocculation threaten environmentally benign, also require high water and energy consumption. To reduce these issues associated with using chemical-based reagents, biodegradable dispersants and flocculants obtained from natural sources (*e.g.*, microorganisms, plants, animals) have been proposed.^12,13^ Application of various types of these “biopolymers” as kaolinite clay flocculants has been comprehensively researched.^14–19^ A few studies have reported the effectiveness of various biopolymers to improve flotation and flocculation processes in mineral processing. Huang (2012) found that chitosan (the amine-based polysaccharide) could depress chalcopyrite (copper ore) selectively in the flotation of copper–lead ores.^20,21^ Li *et al.* (2015) and Peng *et al.* (2012) also investigated the chitosan increased the recovery of galena at pH 3–5 as a depressant of chalcopyrite.^22,23^ Li *et al.* (2015) found that chitosan depressed both molybdenite and chalcopyrite in single mineral flotation at pH 6.^22^ Several research studies have examined the effectiveness of the lignosulfonate as a dispersant of kaolinite clay minerals in gold, coal and copper flotation.^15,24^ Liu (2018) also found that lignosulfonate improved the grade of gold ore by dispersing kaolinite particles at pH 9.^25^ Molatlhegi *et al.* (2017) investigated chitosan as a flocculant of kaolinite particles showed a positive effect on settling of kaolinite particles at pH range 3–9.^18^ Y. C. Ho (2010)^12^ considered the effect of pectin for flocculation of kaolinite particles, and it was found that pH 3 was the optimum treatment condition for pectin flocculating activity.^26^ Bonilla (2015–2017)^14^ studied the effectiveness of proteins (protamine and lysozyme) on the dewatering of pulp and paper mill biosludge and found that lysozyme showed higher flocculating activity.^14,27,28^ Previous studies only focused on kaolinite clay particles and the potential of biopolymers to mitigate other types of phyllosilicates in flotation and dewatering has not been evaluated. More importantly, the main mechanism of interaction between biopolymers and clay surfaces is not yet well understood and has not been considered yet.

**Fig. 1:**
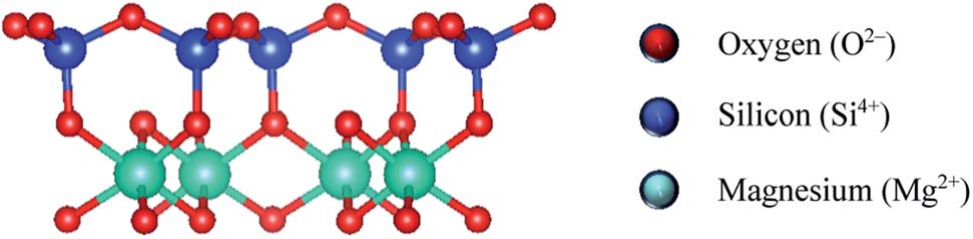
Serpentine 1:1 structure: silicon tetrahedral layer joined to magnesium octahedral layer.^21–23^

The present research, for the first time, focuses on the use of polysaccharide- and protein-based biopolymers to mitigate the negative effects of a non-swelling serpentine phyllosilicate (Mg_3_Si_2_O_5_(OH)_4_) *via* zeta potential measurements, flotation, settling, turbidity, and adsorption isotherm using total organic carbon analyzer. Serpentine is a gangue mineral that often presents in high contents in pentlandite (nickel ore) processing circuits. Serpentine is electrostatistically anisotropic and has a 1:1 structure consisting of Mg^2+^ ions coordinated with oxygen and hydroxyl ions in an octahedral sheet, surrounded by Si^4+^ ions coordinated with oxygen in a tetrahedral basal plane (Fig. 1).^30–32^ The Mg basal plane has positive charge at pH < 10, whereas the edge and Si basal plane are negatively charged at pH 7 and 10.^33–37^

## 2. Materials and methods

### 2.1. Materials

High-purity serpentine was obtained from Minerals Unlimited (Ridgecrest, CA, USA). Pentlandite ((Fe,Ni)_9_S_8_) from Voisey's Bay Mine (Labrador, Canada) was used in microflotation experiments. X-ray diffraction analysis of serpentine and pentlandite samples indicated that they were of high purity (<85% impurities). The mean particle size of serpentine was less than 10 μm as determined by a Partica LA-960 laser diffraction particle size analyzer (HORIBA, Toronto, ON, Canada). For zeta potential measurements, a 0.2 wt% (mass serpentine/volume 18 MΩ deionized (DI) water) serpentine suspension was prepared. For other tests, a 10 wt% serpentine suspension was prepared and adjusted to pH 7 and 10 using 0.1 M hydrochloric acid (HCl), 0.1 M sodium hydroxide (NaOH), or soda ash (Na_2_CO_3_) from Sigma Aldrich Canada. Six commercially available polysaccharides and protein biopolymers from various sources and with differing charge characteristics and molecular weights were purchased from Sigma Aldrich (Table 1). Their zeta potentials and flocculating performance were compared to Brennfloc polyacrylamide (PAM) from Brenntag (Toronto, ON), which is commonly used as a flocculant in wastewater treatment.

**Table 1:** Polymer properties

Polymer	Sourced from	Molecular weight (g mol^−1^)	Type
**Cationic**			
Protamine	Salmon	1523	Protein-based biopolymer
Lysozyme	Egg whites	14 307	Protein-based biopolymer
Chitosan	Chitin	190 000–310 000	Polysaccharide-based biopolymer

**Anionic**			
Alginic acid	Brown algae	240 000	Polysaccharide-based biopolymer
Pectin	Apples	1094	Polysaccharide-based biopolymer
Lignin	Wood	10 000	Polyphenolic biopolymer
Polyacrylamide	Free radical polymerization of acrylamide	1.5 × 10^7^	Synthetic linear polymer

### 2.2. Zeta potential measurements

The zeta potentials of 0.2 wt% serpentine suspensions in the absence (control) and presence of 0.5 wt% (mass of polymer/mass serpentine minerals) polymers (Table 1) (the optimum dosage for biopolymers found by Molaei *et al.* 2022 ^29^) at pH 3, 7, 8.5, and 10 were measured in triplicate using a ZetaCompact Z9000 zetameter (CAD Instruments, Les Essarts le roi, France) with 10 mM KCl as the background electrolyte solution.

### 2.3. Microflotation of pentlandite ores

Triplicate microflotation tests were performed in 50 mL glass Hallimond flotation cells. In a beaker, 2.5 g each of serpentine and pentlandite were dispersed in 45 mL DI water by magnetic stirring for 30 min. The pH was adjusted to 10 with soda ash, then 0.5 wt% of a given polymer was added and conditioned for 2 min prior to addition of 60 g t^−1^ potassium amyl xanthate collector (Flottec, Booton, NJ, USA). The collector was conditioned for 3 min, then 40 g t^−1^ methyl isobutyl carbinol frother (Flottec) was added for a 2 min conditioning period. Controls were prepared the same way with no biopolymer addition. The dispersed pulp was poured into the microflotation cell at 400 rpm impeller speed. The froth was collected for 30 s. Concentrates and tailings were collected, vacuum filtered through a 0.2 μm pore size filter, weighed, and oven-dried at 75 °C for 24 h to be prepared for inductively coupled plasma (ICP) analysis to measure component elements.

### 2.4. Settling tests

Triplicate column settling tests were conducted in 500 mL graduated cylinders using 10 wt% serpentine suspensions (the initial volume of the slurry was 350 mL). The pH was adjusted to 7 and 10 and 0.5 wt% biopolymer or PAM (Table 1) was added for the treatments (although this dosage is high for PAM because of its high viscosity, the purpose is only to compare the settling results of biopolymer with PAM at the same condition); no reagents were added for the controls. The effect on flocculating activity of 0.001 M CaCl_2_ (Ca^2+^ as a divalent cation coagulant) without or in combination with the polymers (PAM and biopolymers) was also investigated. Cylinders were sealed and slowly inverted five times by hand to disperse and homogenize serpentine particles in the solution. The mudline—solid/liquid interface—height was recorded immediately and every 5 min over a 120 min period. After 30 min of each settling test, 15 mL of supernatant was withdrawn with a syringe, and turbidity was measured using a Hach (London, ON, Canada) 2100Q0 turbidity meter.

### 2.5. Adsorption isotherms

To quantify the mass of water-soluble biopolymers (protamine, lysozyme, and pectin) adsorbed on serpentine particles, total organic carbon (TOC) was used to measure the concentration of polymers on triplicate samples with a TOC-VCPH/CPN analyzer (Shimadzu Corporation, Kyoto, Japan). In this procedure, all forms of carbon in solution are converted to CO_2_ by combustion.^38,39^ The TOC apparatus was first calibrated using standard solutions of the three biopolymers. Each test used 20 mL of 10 wt% serpentine suspension that was adjusted to pH 7 or 10, conditioned with 500–10 000 mg L^−1^ biopolymer, mixed at 150 rpm for 48 h to achieve an equilibrium state, and centrifuged at 3000 rpm for 5 min. The supernatant was withdrawn and filtered through a 0.2 μm pore size syringe filter to yield a clear solution. The mass of biopolymer adsorbed on the serpentine surface (*q*, mg g^−1^) was calculated using the following equation.
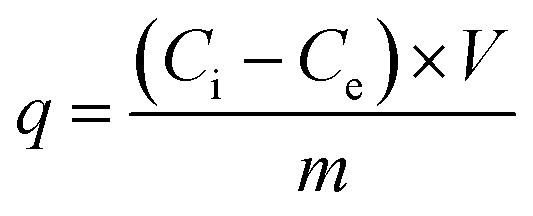
where *C*_i_ and *C*_e_ are the initial and equilibrium TOC concentrations in the supernatant, respectively (mg L^−1^); *V* is the total volume of the serpentine suspension (L); and *m* is the mass of serpentine in the suspension (g).

## 3. Results and discussion

### 3.1. Modification of serpentine surface charge by PAM and biopolymers

**Fig. 2:**
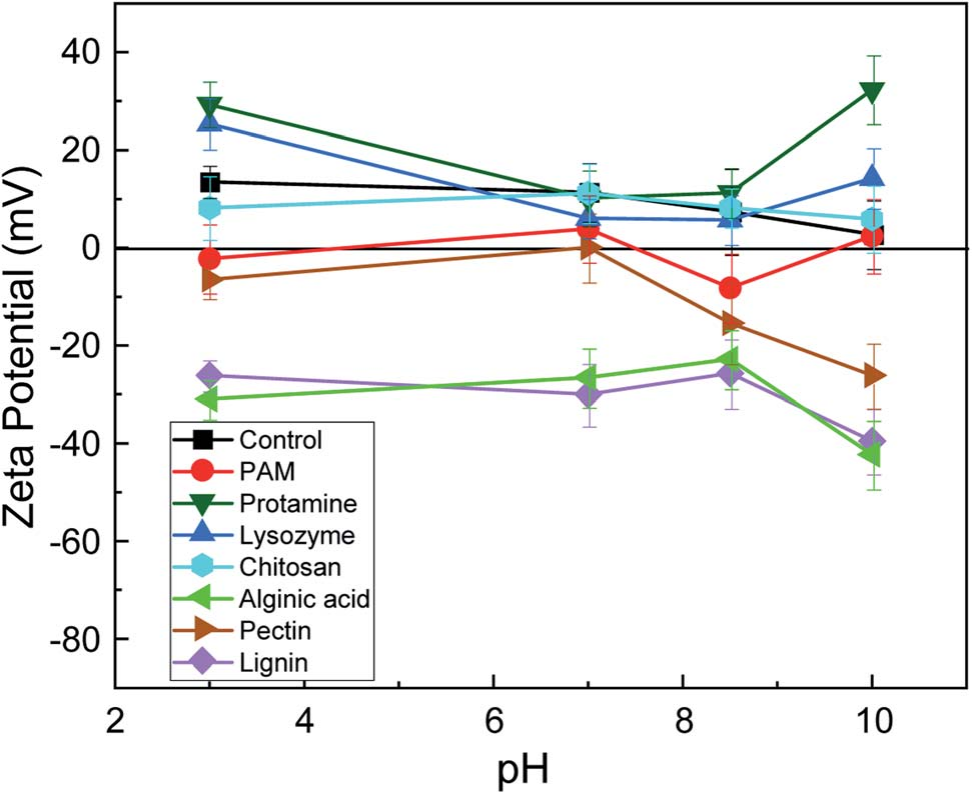
Mean (±standard deviation) zeta potential of triplicate 0.2 wt% serpentine suspensions at pH 3, 7, 8.5, and 10 without (control) and with addition of polyacrylamide (PAM) and six biopolymers.

The mean zeta potential of serpentine surfaces in the control was positive from pH 3 to 10 (Fig. 2), suggesting that the Mg basal plane dominates the surface chemistry. Addition of cationic biopolymers (protamine, lysozyme, and chitosan) had little effect on the zeta potential, except at pH 10 where protamine increased the surface charge of serpentine, indicating strong adsorption of this biopolymer to the serpentine surface. Addition of anionic polymers (alginic acid, pectin, lignin, and PAM) lowered the zeta potential. For alginic acid and lignin, the surface charge reversed from positive to negative. Pectin lowered the zeta potential to near zero at pH 3 and 7 and rendered it negative at pH 8.5 and 10. Thus, pectin may have the potential to act as a switchable biopolymer, flocculating serpentine at pH 7 (the target pH for tailings dewatering) and dispersing it at pH 10 (the target pH for nickel ore flotation).

The surface charge and functional groups of biopolymers and serpentine drive the adsorption process. The repulsive force between cationic biopolymers and the positive surface charge of the serpentine Mg basal plane means that hydrogen bonding between amine groups (NH_2_) in the backbone of cationic biopolymers and the silanol (Si–O–H) groups in the structure of serpentine drive adsorption. The mechanism of adsorption of the anionic biopolymers alginic acid, pectin, and lignin is likely charge neutralization and/or hydrogen bonding between C=O groups of biopolymers and OH groups of serpentine.^29^

### 3.2. Biopolymers as dispersants in flotation

**Fig. 3:**
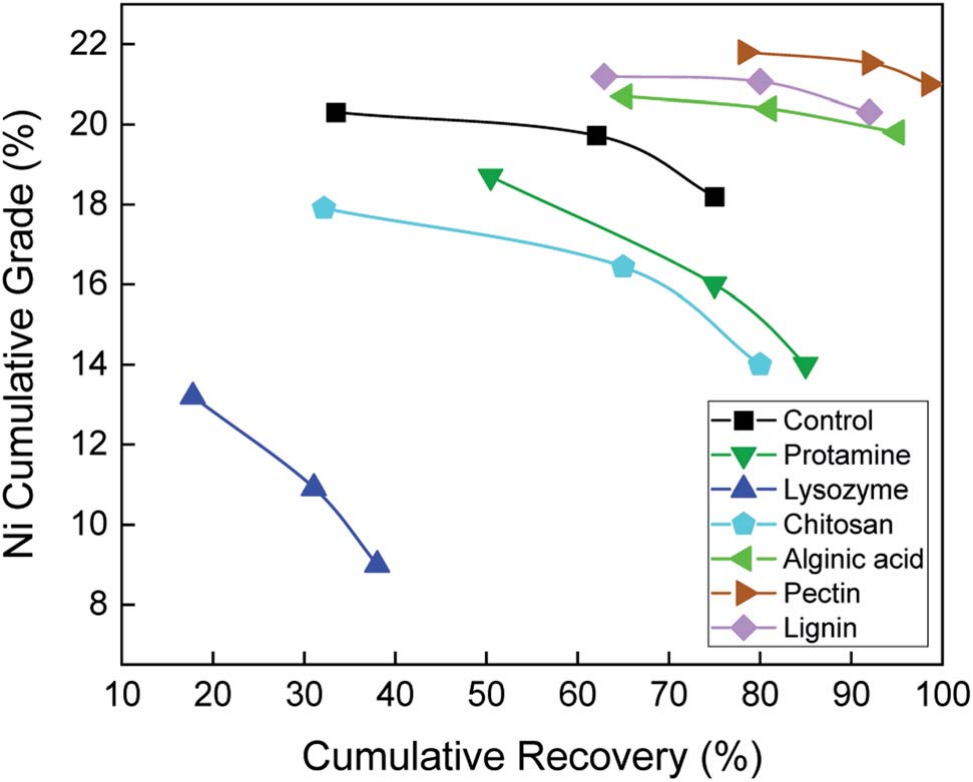
Nickel grade-recovery curves from microflotation tests on 10 wt% (1 : 1 mass ratio) pentlandite–serpentine mixtures without (control) and with the addition of six biopolymers at pH 10.

Pectin dramatically improved flotation of Ni from pentlandite at pH 10 (Fig. 3): Ni grade and recovery (21 and 98.5%, respectively) were higher than the control (18.2 and 75%, respectively). Alginic acid and lignin also improved pentlandite floatability compared to the control, whereas protamine, lysozyme, and chitosan reduced flotation performance. These results were not surprising because the zeta potential results suggested that pectin would be an effective dispersant at pH 10, where the highly negative surfaces of serpentine, especially the silica and edge faces, and negative charge of the biopolymer would contribute to serpentine dispersion. Carboxyl (COOH) functional groups in the polysaccharide backbones of pectin, alginic acid, and lignin could be ionized into COO^−^, which would increase the repulsive forces,^29^ hence improving Ni grade and recovery. The microflotation results show that floatability of pentlandite depends on the polymer and surface charge of serpentine at pH 10.

### 3.3. Settling behavior

**Fig. 4:**
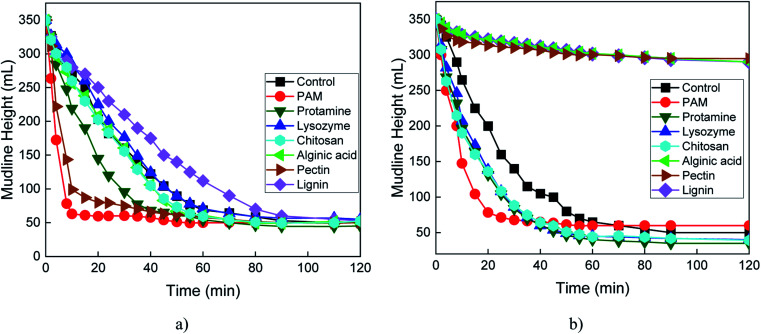
Mudline height *versus* time for 10 wt% serpentine suspensions without (control) or with 0.5 wt% polyacrylamide (PAM) and six biopolymers at (a) pH 7 and (b) pH 10.

In the controls, the sediment/water interface (mudline) height declined at a similar rate at pH 7 (Fig. 4a) and pH 10 (Fig. 4b) over the 2 h settling test. At pH 7, the settling behaviors of serpentine suspensions augmented with lysozyme, chitosan, and alginic acid were similar to the control (Fig. 4a). This is surprising because lysozyme and chitosan are cationic and should have adsorbed to the negative surfaces of serpentine to promote settling at pH 7. Settling was greatly enhanced by addition of PAM, protamine, and pectin, as indicated by a lower mudline height. At pH 7, the Mg basal plane of serpentine is positively charged, whereas the edge and Si basal plane are negatively charged. Weak adsorption between protamine and serpentine surfaces could have occurred *via* charge neutralization because protamine is highly positive at pH 7 due to protonation of amine groups (NH_3_^+^) that have a p*K*_a_ of ∼12.5.^40^ Adsorption of PAM and protamine also occurs *via* hydrogen bonding between NH_2_ groups on the polymer and hydroxyl groups (OH) of Si–OH and Mg–OH at the edge surface of serpentine. Rapid settling of serpentine in the presence of protamine implies that charge neutralization and hydrogen bonding occur simultaneously.^29^ The negative surface charges of carboxyl groups on pectin (p*K*_a_ < 4)^41^ would be attracted to the positively charged Mg basal plane by charge neutralization, resulting in flocculation and settling.

At pH 10 and compared to the control, settling was improved the most by PAM, followed by the cationic biopolymers (Fig. 4b). At pH 10, the Mg basal plane of serpentine has a surface charge near zero, whereas the edge and Si basal plane are negatively charged. Lysozyme (p*K*_a_ ∼ 10.5)^42^ and chitosan (p*K*_a_ ∼ 6.1–7)^43^ are almost neutral, and only when the silica and edge surfaces of serpentine were highly negatively charged did these biopolymers promote settling of serpentine. Settling was hindered by pectin. Pectin was repelled by all the planes, resulting in effective dispersion. The flocculating activity of pectin at pH 7 and dispersing activity at pH 10 suggest pectin is a good candidate as a switchable biopolymer in serpentine processing.

**Fig. 5:**
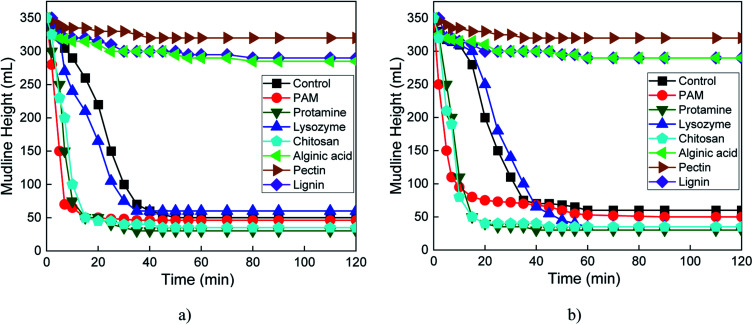
Mudline height *versus* time for 10 wt% serpentine suspensions without (control) or with 0.5 wt% biopolymer or polyacrylamide (PAM) and 0.001 M CaCl_2_ at (a) pH 7 and (b) pH 10.

Unlike pectin alone (Fig. 4a), addition of 0.001 M CaCl_2_ with pectin inhibited settling of serpentine at pH 7 (Fig. 5a). This can be explained by adsorption of Ca^2+^ on the negatively charged pectin surface preventing pectin adsorption on the positively charged surface serpentine.^44–46^ At pH 7 (Fig. 5a) and 10 (Fig. 5b), CaCl_2_ promoted serpentine settling in combination with chitosan and protamine, had a negligible effect with lysozyme, and no effect with PAM, alginic acid and lignin.

**Fig. 6:**
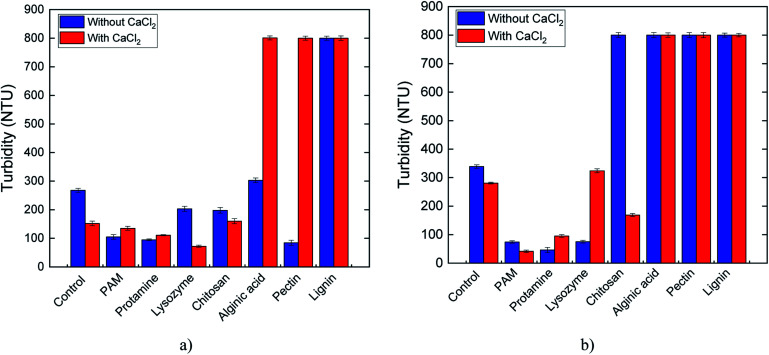
Turbidity measurements of collected supernatant from settling tests for serpentine with and without CaCl_2_ coagulant at (a) pH 7 and (b) pH 10.

At pH 7, the turbidity of serpentine suspensions without CaCl_2_ was the lowest for pectin and the highest for lignin and alginic acid (Fig. 6a), indicating strong particle aggregation and dispersion, respectively. The strong inhibition of the flocculation ability of pectin by CaCl_2_ is evident in the nearly 10-fold higher turbidity. Flocculation was less dramatically reduced in the PAM and protamine treatments. CaCl_2_ increased aggregation for the control and the lysozyme- and chitosan-treated suspensions. At pH 10, PAM, lysozyme, and protamine were excellent flocculants when used alone, whereas in the presence of CaCl_2_, PAM, protamine, and chitosan enhanced flocculation (Fig. 6b). Lignin was a very poor flocculant at both pH 7 and 10, with no effect by CaCl_2_. It is evident that the interactions between biopolymers and Ca^2+^ at the surface of serpentine are complex and require further investigation to understand the underlying mechanisms.

### 3.4. Adsorption isotherm by TOC

**Fig. 7:**
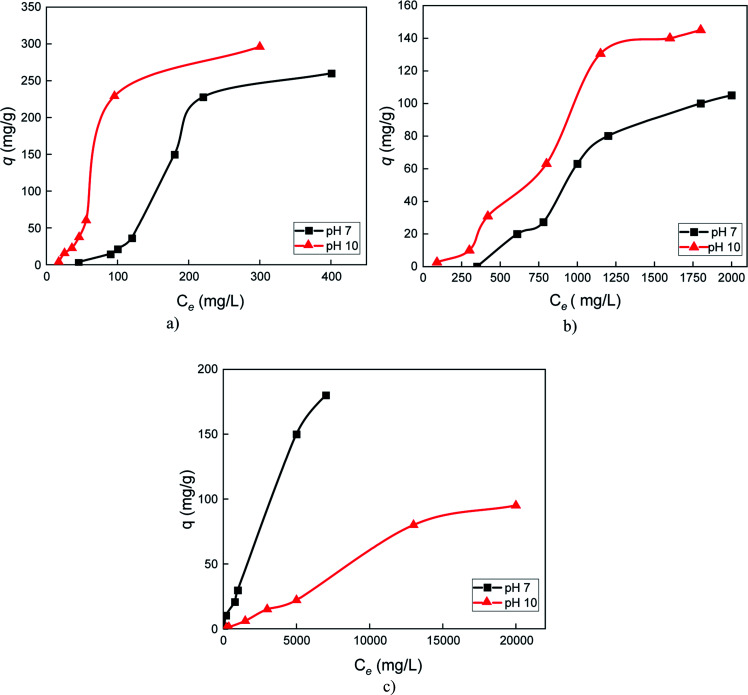
Adsorption isotherms on serpentine at pH 7 and 10 for (a) protamine, (b) lysozyme, and (c) pectin; *q*: mass of biopolymer adsorbed on the serpentine surface; *C*_e_: equilibrium total organic carbon concentrations in the supernatant.

Serpentine had strong adsorption affinity with the cationic biopolymers protamine (Fig. 7a) and lysozyme (Fig. 7b), particularly at pH 10 (higher values for *q*), possibly due to formation of large aggregates between serpentine particles. Adsorption also increased with biopolymer dosage, suggesting that adsorption sites on serpentine were not saturated. Hydrogen bonding between NH_2_ and COOH groups of proteins and hydroxyl groups of serpentine and/or electrostatic interaction between the cationic biopolymers and the negatively charged surface of the serpentine edge and Si basal planes at pH 7 contributed to adsorption.^29^ Pectin strongly adsorbed on the serpentine surface only at pH 7 and no equilibrium was observed (Fig. 7c). There might be a strong affinity between pectin and serpentine, in agreement with the settling test results in (Fig. 4a), which may be attributed to electrostatic interaction between anionic pectin and the positively charged surface of Mg basal plane of serpentine.

## 4. Conclusions

This work investigated how six biopolymers with different surface charge and characteristics affected the flocculation and dispersion of serpentine particles at pH 7 and 10 and compared them to PAM, a commonly used synthetic polymer. At pH 7, the zeta potential of pectin approached neutrality, which enhanced the settling rate of serpentine more than did the other biopolymers. At pH 10, however, pectin behaved as a strong dispersant, as indicated by low settling and high turbidity. Protamine also improved serpentine settling at pH 7, though not as dramatically as pectin. Protamine, lysozyme, and chitosan improved the settling rate of serpentine compared to the control at pH 10. Isotherm adsorption experiments confirmed strong adsorption of pectin on serpentine at pH 7. The main driving mechanism for cationic biopolymers in the presence of negatively charged particles is electrostatic attraction. Divalent cations in the solution made bridges between the negative surface of serpentine and anionic biopolymers. Charge neutralization also played an important role in the adsorption of pectin with serpentine. It can be concluded that pectin acted as a switchable biopolymer for serpentine: it showed flocculating activity at the dewatering pH of 7 and dispersing activity at the flotation pH of 10. Results of this study are applicable not only in mineral processing but also in other industries that have clay issues; pulp and paper mill, wastewater treatment, food production, cement production and ceramics operations. A deeper understanding of biopolymer adsorption mechanism using techniques such as atomic force microscopy (AFM) and quartz crystal microbalance with dissipation (QCM-D) is necessary to apply pectin in mineral processing operations and will remain the subject of our future work.

## Conflicts of interest

There are no conflicts to declare.

## Supplementary Material
